# A Photocleavable Amphiphilic Prodrug Self-Assembled Nanoparticles with Effective Anticancer Activity In Vitro

**DOI:** 10.3390/nano9060860

**Published:** 2019-06-05

**Authors:** Ji Chen, Guotao Li, Qihong Liu, Yan Liang, Miaochang Liu, Huayue Wu, Wenxia Gao

**Affiliations:** 1 College of Chemistry and Materials Engineering, Wenzhou University, Wenzhou 325027, China; 16451282234@stu.wzu.edu.cn (J.C.); chemliguotao@sina.com (G.L.); qihong.liu@pharmaron-bj.com (Q.L.); huayuewu@wzu.edu.cn (H.W.); 2 Department of Pharmaceutics, School of Pharmacy, Qingdao University, Qingdao 266021, China; liangyan@qdu.edu.cn

**Keywords:** prodrug, photocleavable, stimuli-responsive release, methotrexate, chemotherapy

## Abstract

Accelerating degradation of prodrug is an effective strategy for improving the pharmacological action. A photocleavable amphiphilic prodrug of methotrexate-coumarin derivative-PEG conjugates (MTX-AMC-PEG) with photo-triggered breakage to release clinical drug under laser irradiation was fabricated and self-assembled into nanoparticles for chemotherapy. The nanoparticles exhibited good intracellular uptake and excellent photolysis release of MTX, which resulted in efficient anticancer activity in vitro with laser irradiation. This research provides a way to fabricate photocleavable prodrug nanoparticles with stimuli-triggered drug release behavior.

## 1. Introduction

Chemotherapy remains one of the major strategies for cancer treatment, but unfortunately, there are several limitations in chemotherapy. For example, many chemotherapeutics are hydrophobic with poor bioavailability, and most of them cannot be delivered and accumulated in tumor sites, which results in serious side effects [1,2,3]. Fabrication of prodrugs is reported as an effective way to resolve the limitations and minimize the side effects [4,5]. However, with the rapid blood/renal clearance of prodrugs, there remains a problem of how to increase their bioavailability [6]. Nanovehicles including biocompatible polymers, liposomes, and inorganic nanoparticles have been developed and employed as drug carriers [7,8,9]. Recently, nanoparticles self-assembled from prodrugs with hydrophobic drugs and hydrophilic moieties became a new strategy to prepare nano-drug delivery systems. Yan and coworkers synthesized a prodrug of amphiphilic anticancer drug–drug conjugate, the prodrugs self-assembled nanoparticles and their cancer-therapeutic efficacy was improved dramatically [10]. Wang reported disulfide-linked doxorubicin prodrugs-based nanoparticles to enhance the cellular uptake level and antitumor activity [11]. These low-molecular-weight prodrugs-based DDS not only improved drug loading, but also enhanced the anti-tumor efficiency. However, defects of nano-prodrug DDS, such as slow drug release which impacts the curative effect, still exist. As the prodrug conjugates exert their pharmacological actions only after the conjugates are transformed into free drugs, [12] endogenous stimuli such as pH, hydrolysis, oxidation reduction, and enzymes are usually used to trigger the cleavage of conjugates [13,14,15]. The effects of most endogenous stimuli are slow in transformation, thus, some external stimuli are involved to accelerate the release rate, such as light, magnetic force, ultrasound, and so on [16,17,18]. However, these non-biological stimuli signals are usually loaded in nanocarriers to destroy the architectures of self-assembled nanoparticles, which are not helpful to accelerate the degradation or bond cleavage of prodrugs [19]. Therefore, the prodrugs are expected to possess functional moieties sensitive to external stimuli.

Although coumarin derivatives were extensively reported as photo-cleavable moieties for photo-responsive study, few researchers focused on the coumarin derivatives as photo-responsive units in the fabrication of prodrug self-assembled nanoparticles for chemotherapy. In this paper, a photo-triggered hydrolysis of an amphiphilic prodrug conjugate was developed for light-induced release of anticancer drugs. Methotrexate (MTX) was selected as the model drug, taking advantage of the folic acid antagonist, it was used in treating malignant tumors by inhibiting dihydrofolate reductase to block DNA synthesis [20]. A coumarin derivative was selected as the photo-controllable moiety to induce the bond breakage under laser irradiation, the light-response was activated by UV or two-photon fluorescence [21,22]. The coumarin derivative of 7-[bis (carboxymethyl) amino]-4-(hydroxymethyl) coumarin (AMC) was synthesized, MTX was bonded to the coumarin derivative to fabricate the light-responsive prodrug, and PEG2000 was immobilized on the prodrug to receive the amphiphilic polymers (MTX-AMC-PEG conjugate) and self-assembly into nanoparticles. The aim of PEGylated nanoparticles was to prolong the circulation of nano-DDS and improve the passively targeting efficacy via the enhanced permeability and retention (EPR) effect [23].

## 2. Experimental Section

### 2.1. Materials and General Techniques

7-Amino-4-methylcoumarin (AMC) and selenium dioxide (SeO_2_) were obtained from Shanghai Aladdin Chemistry Co. Ltd. (Shanghai, China). Trifluoroacetic acid (TFA), tertbutyl bromoacetate, 1-(3-dimethylaminoprapyl)-3-ethylcarbadiimidehydrochloride (EDC), methotrexate (MTX), and *N*-hydroxysuccinimide (NHS) were bought from Shanghai Energy Chemical Co. Ltd. (Shanghai, China). mPEG-NH_2_ (Mw = 2200 g/mol) was purchased from Xi’an Ruixi Biotechnology Co., Ltd. (Xi’an, China). All the solvents were purchased from Energy Chemical Co. Ltd. (Shanghai, China) and purified before use. Dulbecco’s modified Eagle’s medium (DMEM), 100 x mycillin, fetal bovine serum (FBS) and cell counting kit-8 (CCK-8) were purchased from HyClone Inc. (South Logan, UT, USA) and used for cytotoxicity test.

The compounds were characterized by NMR spectroscopy (Bruker-500 spectrometer, Faellanden, Switzerland, 500 MHz for ^1^H NMR, 125 MHz for ^13^C NMR) and mass spectrometric analysis (Bruker microTOF-QII HR-MS analysis, Faellanden, Switzerland). The morphology and particle size distribution of nanoparticles were characterized by scanning electronic microscopy (SEM, NovaNanoSEM200 scanning electron microscope, Hillsboro, OR, USA), transmission electron microscope (TEM, JEM-2100F transmission electron microscope, JEOL, Tokyo, Japan), and Dynamic Light Scattering (DLS) (Malven Nano S Zetasizer, Malven, UK). Photocatalytic bond-breaking and drug release process were recorded on HPLC (Agilent 1120 using inertsil ods-sp C18 chromatographic column, Santa Clara, CA, USA), UV spectra (Shimadzu UV2501PC, Kyoto, Japan) and Fluorescence (HITACHI F-7000, Tokyo, Japan). 

### 2.2. Synthesis of MTX-AMC-PEG Conjugate

**Scheme 1:**
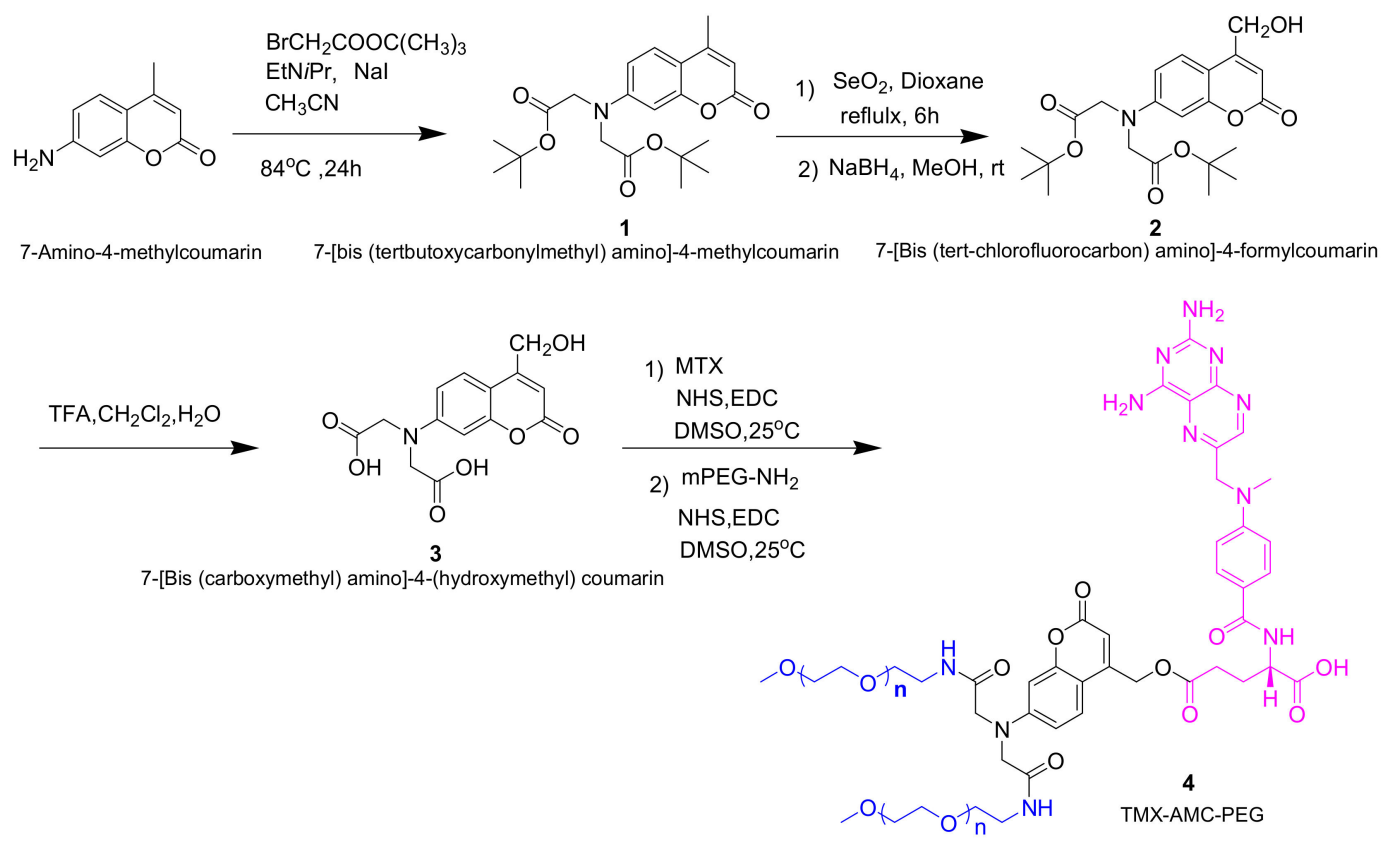
The synthesis of MTX-AMC-PEG conjugate.

The synthetic process of MTX-AMC-PEG conjugate is shown in Scheme 1, and the characterization of the compounds were available in Figures S5–S9.

#### 2.2.1. Synthesis of Compound **1**: 7-[bis (tertbutoxycarbonylmethyl) amino]-4-methyl-coumarin

AMC (15 mmol, 2.63 g), diisopropylethylamine (10.27 mL, 60 mmol), bromoacetic acid tert-butyl ester (14.69 mL, 90 mmol), and NaI (2.25 g, 15 mmol) in 90 mL CH_3_CN were stirred at 85 °C for 24 h. The mixture was filtered, and the solvent was concentrated in vacuum. The residue was dissolved in ethyl acetate, thrice-washed with saturated brine, and dried with anhydrous sodium sulfate. The solvent was evaporated in vacuum to obtain a yellow oil. The crude product was purified by silica column chromatography (eluent: EA/PE = 1/6) to obtain compound **1** (2.05g, 5.08 mmol, 34% yield).

Yellow solid, M.p: 151.1 °C. ^1^H NMR (CDCl_3_, TMS): δ = 1.47 (s, 18 H), 2.35 (s, 3 H), 4.06 (s, 4H), 6.01 (s, 1 H), 6.45 (d, 1 H), 6.53 (dd, 1 H), 7.42 (d, 1 H). ^13^C NMR (CDCl_3_, TMS): δ = 18.42, 28.05, 54.37, 82.40, 99.09, 108.93, 110.40, 111.22, 125.56, 150.83, 152.85, 155.46, 161.43, 169.00.

#### 2.2.2. Synthesis of Compound **2**: 7-[Bis (tert-chlorofluorocarbon) amino]-4-formylcoumarin

Compound **1** (1.0 g, 2.5 mmol) was dissolved in p-xylene (30 mL), SeO_2_ (0.747 g, 5 mmol) was added, and the mixture was stirred at 145 °C for 8 h. The black selenium was filtered, the solution was concentrated to obtain brown residual oil. The crude product in methanol was reduced by sodium borohydride (0.14 g, 3.7 mmol) at 25 °C for 2 h. The suspension was neutralized with hydrochloric acid (1 M) and diluted with distilled water. The mixture was concentrated in vacuum and extracted with CH_2_Cl_2_. The organic phase was washed with water and brine, dried over anhydrous sodium sulfate and evaporated in vacuum. The crude product was purified by column chromatography (PE/EA = 4/1) to obtain 0.43 g (1.0 mmol, 42% yield) of compound **2**, brown solid, M.p:174.8 ℃.^1^H NMR (CDCl_3_, TMS): δ = 1.49 (s, 18H), 3.49 (s, 1H), 4.05 (s, 4H), 4.64 (s, 2H), 6.30 (s, 1H), 6.42(s,2H), 7.17 (s, 1H). ^13^C NMR (CDCl_3_, TMS): δ = 28.03, 54.20, 60.31, 82.62, 99.00, 106.77, 108.48, 109.05, 124.18, 150.94, 155.07, 155.27, 166.22, 169.22.

#### 2.2.3. Synthesis of Compound **3**: 7-[Bis (carboxymethyl) amino]-4-(hydroxymethyl) Coumarin

Compound **2** (0.4878 g, 1.1 mmol) was stirred in a mixture (20 mL) of TFA/H_2_O/CH_2_Cl_2_ (74: 1: 25) at 25 °C for 3 h. The solution was concentrated, the residue was dissolved in the mixed solvents of CH_3_CN/H_2_O and lyophilized to obtain compound **3** (0.30 g, 93% yield). Brown solid. M.p:186 °C.^1^H NMR (DMSO-*d*_6_, TMS): δ = 4.22 (s, 4 H), 4.68 (s, 2 H), 5.51 (s, 1 H), 6.15 (s, 1 H), 6.42 (s, 1 H), 6.56 (d, 1 H), 7.47 (d, 1 H), 12.86 (s, 2 H). ^13^C NMR (CDCl_3_, TMS): δ = 52.76, 58.83, 98.04, 104.84, 107.01, 108.89, 125.13, 151.09, 154.89, 156.78, 160.84, 171.37.

#### 2.2.4. Synthesis of Compound **4**: TMX-AMC-PEG

The whole procedure was carried out in the dark. Compound mitoxantrone (40 mg, 0.09 mmol) was dissolved in DMSO (10 mL). EDC (36 mg, 0.18 mmol) was added in the mixture and stirred at 25 °C for 20 min. NHS (60 mg, 0.52 mmol) was added and stirred for another 20 min. Compound **3** (60 mg, 0.20 mmol) was added to the mixture. After stirred for 48 h, EDC (84 mg, 0.44 mmol) and NHS (102 mg, 0.88 mmol) were added orderly. The mPEG-NH2 (0.889 g, 0.45 mmol) was added and stirred at 25 °C for 5 days. The mixture was added dropwise to 150 mL of frozen diethyl ether with stirring and standing at 0 °C for 2 h. The filtered residue was reprecipitated for three times. The residue dissolved in deionized water was dialyzed (dialysis bag, MWCO: 3000D) at room temperature. The 800 mL deionized water outside the dialysis bag was replaced by fresh water every 2 h at first day and every 5 h for the following 4 days. The solution was freeze-dried to obtain product. ^1^H NMR (DMSO-*d*_6_, TMS): δ = 4.20 (s, 2 H), 4.43 (s, 1 H), 4.67 (s, 4 H), 4.80 (s, 2 H), 6.16 (s, 1 H), 6.25 (s, 1 H), 6.40 (m, 1 H), 6.55 (s, 2 H), 6.83 (s, 2 H), 7.45 (s, 3 H), 7.70 (m, 2 H), 8.23 (s, 1 H), 8.58 (m, 1 H). The conjugate was further confirmed by molecular weight of MALDI-TOF-MS analysis (Figure S10), the molecular weight of TMX-AMC-PEG conjugate was about 5500, and the conjugate was grafted by two mPEG-NH_2_.

### 2.3. Preparation and Characterization of MTX-AMC-PEG Nanoparticles

10 mg of MTX-AMC-PEG was dissolved in 1 mL of ethanol. The solution was added dropwise to 10 mL of deionized water with stirring, followed by dialysis against deionized water and concentrated to a final concentration of 1 mg/mL. The size and size distribution of the nanoparticles were measured by DLS and TEM.

### 2.4. In Vitro Drug Release Study

The in vitro release of MTX was performed in PBS (pH = 7.4) at 37 °C. MTX-AMC-PEG was homogenously dispersed in 3 mL buffer solution in dark. At desired time intervals, the samples were treated by 365 nm (5 W, 12 W, 50 W) for different time. At prescribed time, the samples were obtained and measured by HPLC Agilent 1120 using the mixture of methanol (20%) and 0.025 mol/L NaH_2_PO_4_ (80%) at 28 °C with F = 1.0 mL/min. The absorbance of MTX was measured at 306 nm. The release experiments were performed in triplicate, and the results were the average data.

### 2.5. Cellular Uptake

4T1 breast cancer cells were seeded on (diameter = 35 mm) glass dishes with a cell density of 1 × 10^5^ mL^−1^. After incubated for 24 h, the cancer cells were treated with MTX-AMC-PEG nanoparticles at 37 °C for 2, 5, and 12 h. The medium of all samples was removed and the dishes were rinsed with PBS (pH = 7.4). The cells were observed with confocal laser scanning microscopy (Leica TCP SP5, Wetzlar, Germany). MTX-AMC-PEG nanoparticles were excited at 350 nm with the emission at 468 nm.

For flowcytometry study, 4T1 breast cancer cells were seeded on 6-well plates with a density of 2 × 10^5^ cells/well and cultured for 24 h. 4T1 breast cancer cells were incubated with MTX-AMC-PEG nanoparticles at 37 °C for 2, 5, and 12 h. The cells were resuspended in PBS after centrifugation (1000 rpm/min, 5 min) and measured for the fluorescence intensity on a BD FACS Caliburflowcytometer (Beckton Dickinson, Franklin Lakes, NJ, United States).

### 2.6. Cytotoxicity Test and In Vitro Anticancer Activity

The cytotoxicity test and in vitro anticancer activity was assessed with reference to the reported literature [24]. The detailed operating procedures were described in ESI experimental section.

## 3. Results and Discussion

**Figure 1:**
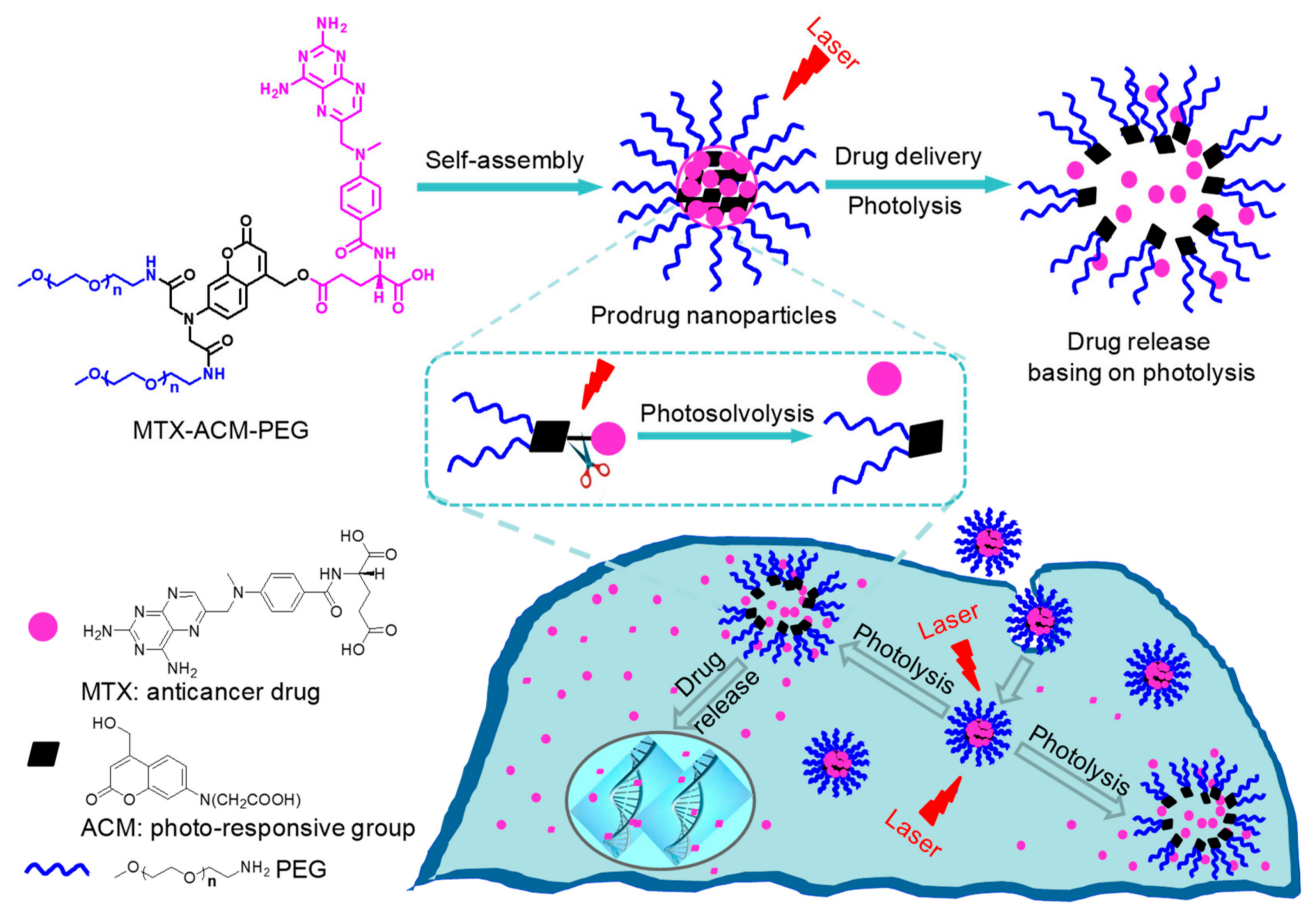
The self-assembly of methotrexate-coumarin derivative-PEG (MTX-AMC-PEG) conjugate prodrug into nanoparticles with photoresponsive release via photosolvolysis.

The synthetic route of photo-sensitive amphiphilic MTX-AMC-PEG conjugate was presented in Scheme 1, and the received products in each step were characterized in Electronic Supplementary Materials. The UV light irradiation activated the photolysis to induce MTX release from the self-assembled nanoparticles as shown in Figure 1. The anticancer activity of photo-sensitive nanoparticles in vitro was studied.

**Figure 2:**
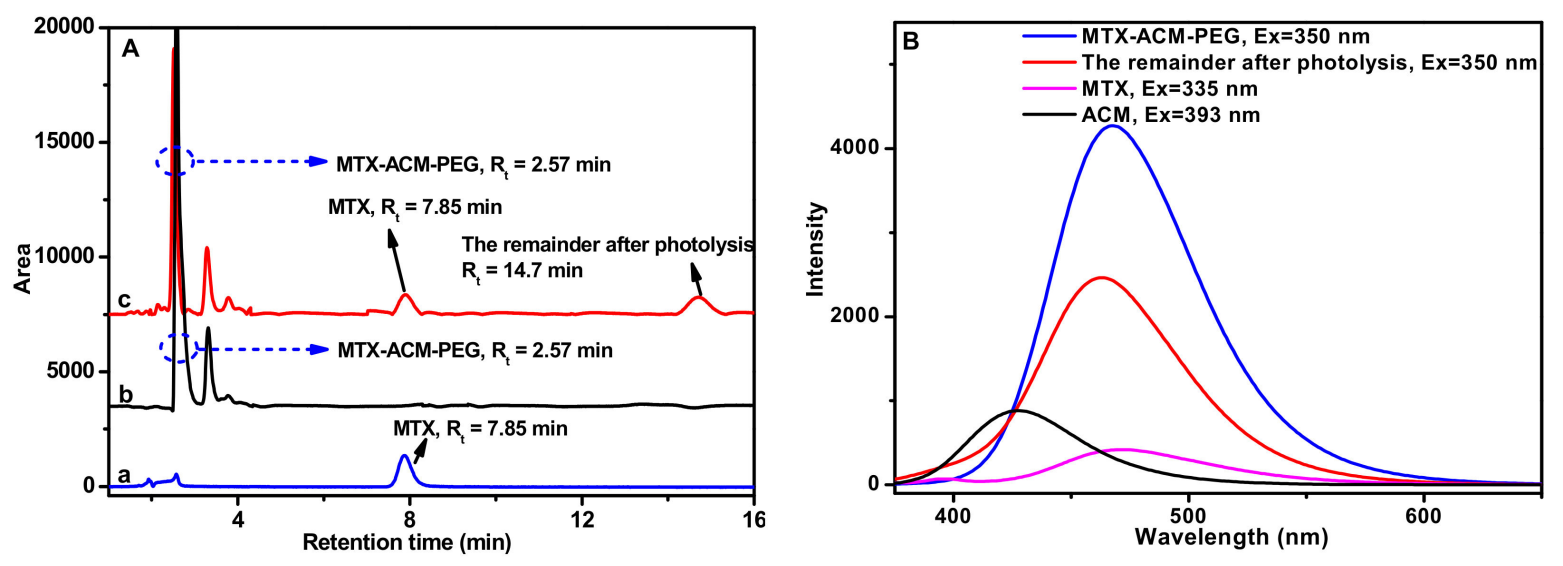
The photosolvolysis process of MTX-AMC-PEG conjugate, (**A**) HPLC spectra of native MTX (**a**), blank MTX-AMC-PEG (**b**) and the products after the photolysis of MTX-AMC-PEG (**c**); (**B**) the fluorescence spectra of native MTX, AMC, blank MTX-AMC-PEG, and the remainder after photolysis in PBS (pH 7.4).

The photo-response of the nanoparticles was investigated, and the photo-triggered release of MTX was showed in Figure 1. After one hour exposure to the laser, the photolysis products were detected by HPLC (Figure 2A), the native MTX was at R_t_ = 7.85 min (Figure 2(Aa)) and MTX-AMC-PEG was at R_t_ = 2.57 min (Figure 2(Ab)), the new peaks at Rt = 7.85 min and Rt = 14.7 min in Figure 2(Ac) were assigned to the MTX by the benzyl ester bond breaking in AMC and another photolysis product with the C-N bond breaking of amino in coumarin unit from the MTX-AMC-PEG conjugate. The photolysis products were further characterized by ^1^H NMR and ^13^C NMR, the MTX-AMC-PEG conjugate had the chemical shifts as below (Figure S1A), ^1^H NMR (DMSO-*d*_6_, TMS): δ =1.73 (2H), 2.01 (2H), 4.19 (s, 2H), 4.43 (s, 1H), 4.67 (s, 4H), 4.80 (s, 2H), 6.14 (s, 1H), 6.25 (s, 1H), 6.40 (m, 1H), 6.55 (s, 2H), 6.83 (s, 2H), 7.45 (s, 3H), 7.70 (m, 2H), 8.23 (s, 1H), 8.58 (m, 1H). The photolysis product at R_t_ = 7.85 min had the chemical shifts as below (Figure S1B), ^1^H NMR (DMSO-*d*_6_, TMS): δ = 1.91 (m, 1H), 2.04 (m, 1H), 3.21 (s, 3H), 2.32 (t, 2H, J = 7.5Hz), 4.34 (m, 1H), 4.79 (s, 2H), 6.68 (s, 2H), 6.83 (d, 2H, J = 8.5 Hz), 7.49 (s, 1H), 7.73 (m, 2H), 8.20 (d, 2H), 8.57 (s, 1H). ^13^C NMR (DMSO-*d*_6_, TMS): δ = 26.06, 30.45, 51.75, 54.51, 99.41, 111.04, 121.31, 128.64, 145.22, 149.00, 150.85, 154.98, 162.50, 166.30, 172.88. The chemical shifts of this product (R_t_ = 7.85 min) was consistent with that of MTX. The MTX-AMC-PEG conjugate exhibited fluorescence due to the chromophore of AMC and MTX. After photolysis, the fluorescence was changed, as showed in Figure 2B. These results demonstrated that the ester bond was broken in AMC moieties, which damaged the structure of nanoparticles and led to MTX release.

**Figure 3:**
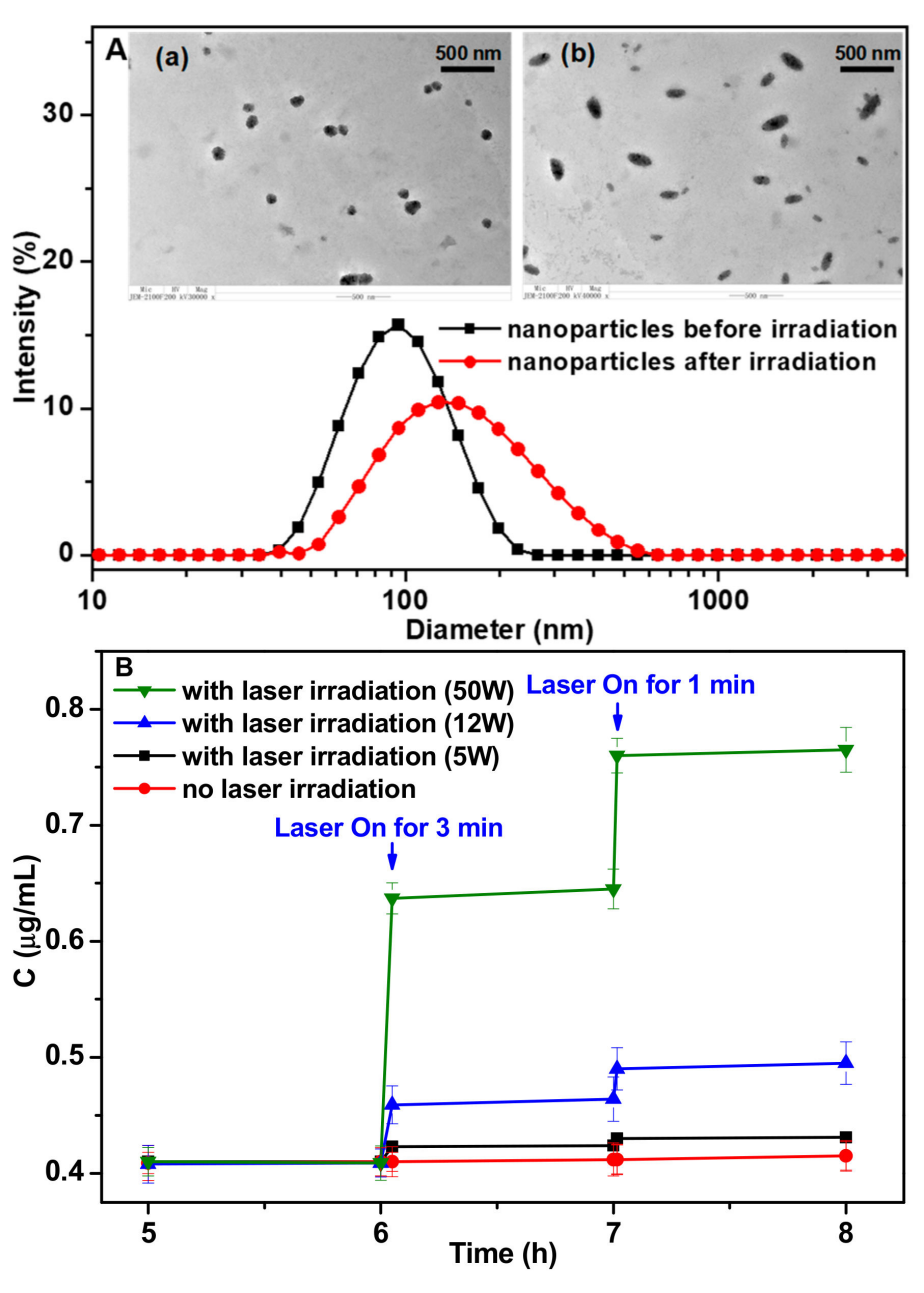
(**A**) DLS results and TEM images (**a**, before irradiation; **b**, after irradiation) of the self-assembled MTX-AMC-PEG nanoparticles; (**B**) The photo-responsive release profiles of MTX from the nanoparticles (1 mg/mL) exposed to laser irradiation (365 nm, 5.0 W, 12 W, 50 W) for 1 min and 3 min in PBS (pH 7.4). Means + SD (n = 3).

The drug release behavior of the self-assembled prodrug nanoparticles was investigated (Figure 1), and the size distributions of the nanoparticles were measured by DLS (Figure 3A). The mean diameter of the nanoparticles was 95.1 ± 1.2 nm, and the TEM image revealed that the nanoparticles were spherical. The nanoparticles were relatively stable in the pH 7.4 and pH 8.5 buffer solutions, and the particle size and morphology remained stable during 50 h dispersed in these buffer solutions. In pH 5.5 buffer solution, the size of nanoparticles gradually became bigger and the particle size distribution became wider (Figure S2). The conjugate was stable and no degradation was observed. The effect of laser irradiation on the morphology and size of nanoparticles was investigated. Great changes have taken place in the morphology and size distribution after long laser irradiation (30 min). The morphology of nanoparticles was changed rugby ball like nanoparticles after 30 min irradiation, and the particle size distribution was widened (Figure 3A). These changes were mainly due to the photoinduced bond breakage, which resulted in the disassociation of nanoparticles structure.

**Figure 4:**
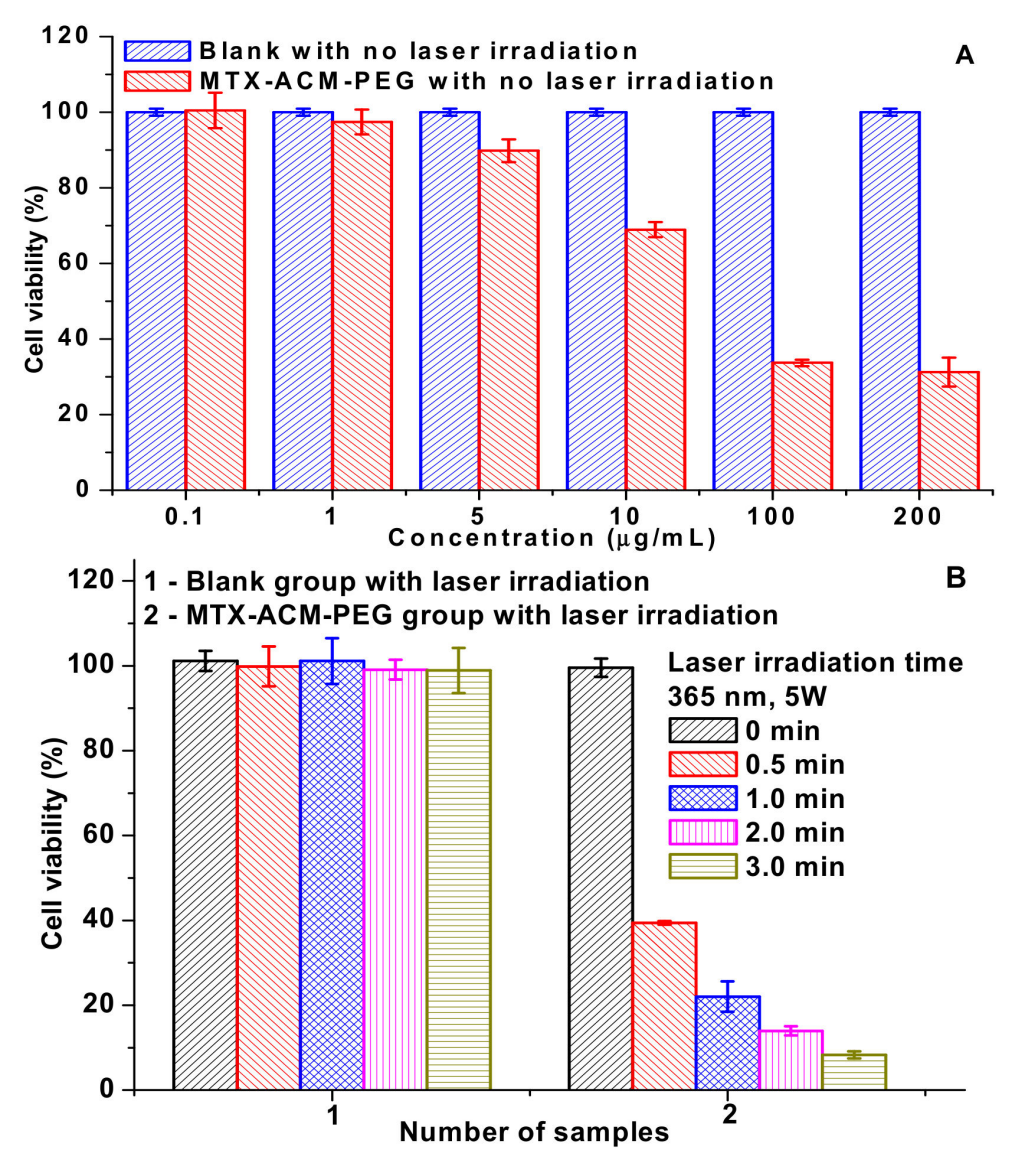

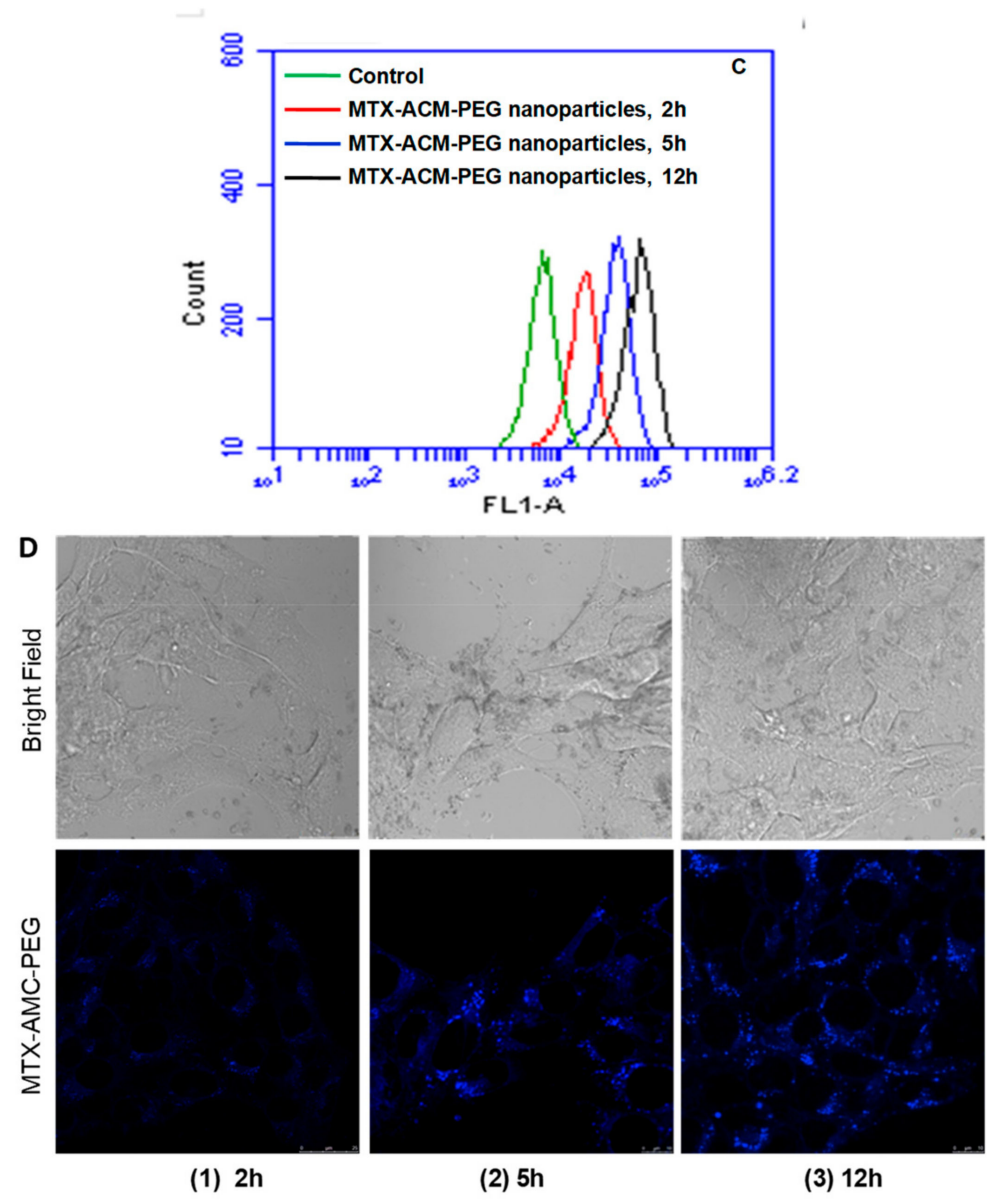
The cytotoxicity of MTX-AMC-PEG nanoparticles to 4T1 breast cancer cells, (**A**) cell viability of 4T1 cancer cells incubated with MTX-AMC-PEG nanoparticles for 48 h; (**B**) cell viability of 4T1 cancer cells incubated with MTX-AMC-PEG nanoparticles (0.4 µg/mL) under 365 nm (5 W) for 0, 0.5, 1.0, 2.0, and 3.0 min. Sample 1 was the control group of blank cells with laser irradiation (without MTX-AMC-PEG nanoparticles), sample 2 was the cells with MTX-AMC-PEG nanoparticles and different time of laser irradiation; (**C**) flow cytometry profiles of 4T1 cells incubated with MTX-AMC-PEG nanoparticles for 2 h, 5 h, and 12 h; (**D**) confocal laser scanning microscopy images of 4T1 cells incubated with MTX-AMC-PEG nanoparticles for 2 h, 5 h, and 12 h.

The release profiles of MTX-AMC-PEG conjugate in PBS (pH 7.4) with or without laser irradiation were also tested (Figure 3B). As long-term laser irradiation would damage cells, the drug release profiles with short irradiation time of 1 and 3 min were investigated (Figure 3B) for the preliminary evaluation to the following in vitro anticancer activity study, and the irradiation time for in vitro cell viability was controlled consistently within 3 min (Figure 4B). The solutions of MTX-AMC-PEG were treated with 365 nm irradiation for 1 and 3 min. Nearly no degradation of MTX-AMC-PEG occurred with irradiation, while the concentration decreased greatly after laser irradiation (Figure S3). Compared with the MTX released from MTX-AMC-PEG nanoparticles without laser irradiation, an increment of 0.014 μg/mL MTX released under 3 min of laser irradiation (5 W) was found, which could indicate a good suppressive effect on the tumor as the IC50 of MTX against 4T1 cells was 0.012 μg/mL (Figure S4). The amount of released drug was enough to reach the effective concentration to kill cancer cells. The irradiation under three laser power (5 W, 12 W, 50 W) was investigated in order to enhance the efficiency of photolysis. To the nanoparticles with the concentration of 1.0 mg/mL (containing 92.98 μg/mL MTX), the drug release was increased nearly fourfold from 0.014 μg/mL to 0.054 μg/mL when the laser power increased from 5 W to 12 W, and the drug release rate increased 17 times to 0.235 μg/mL if the power increased to 50 W. Higher power led to more effective photolysis and the architecture of nanoparticles was more seriously destroyed to result in faster MTX release.

The anticancer activity of MTX-AMC-PEG nanoparticles without laser irradiation was evaluated with 4T1 breast cancer cells (Figure 4A). The cell viability was beyond 90% once the concentration of nanoparticles was lower than 5 µg/mL, the result showed that the prodrug nanoparticles were less toxic to 4T1 breast cancer cells as few MTX was released from the prodrug nanoparticles without irradiation. Once the concentration was higher than 100 µg/mL, the prodrug nanoparticles showed good antitumor activity with 48 h incubation.

To promote the decomposition of prodrug, laser irradiation was added to MTX-AMC-PEG nanoparticles to improve the anti-tumor activity, as shown in Figure 4B. Blank cells were controlled (sample 1) to investigate the effect of laser on cells, and the survival rate was higher than 90% with 1–3 min laser irradiation. Therefore, the short-term irradiation was not obviously toxic to blank cells. The cells viability of cells incubated with MTX-AMC-PEG nanoparticles (0.4 µg/mL) under 365 nm irradiation (5 W) for 0, 0.5, 1.0, 2.0, and 3.0 min (sample 2) were measured. The cytotoxicity was significantly different to the sample 1. The cell viabilities of sample 2 with 0, 0.5, 1.0, 2.0, and 3.0 minutes irradiation were 99.6%, 39.4%, 22.1%, 14.0%, and 8.3%, respectively. A significant difference between the groups was found (*p* < 0.01). It indicated that the MTX-AMC-PEG nanoparticles exhibited excellent anticancer activity under laser irradiation as the irradiation cleaved the bond in the prodrug to destroy the architecture of nanoparticles and MTX was rapidly released from the nanoparticles.

The intracellular uptake of MTX-AMC-PEG nanoparticles was characterized by flow cytometry (Figure 4C) and confocal laser scanning microscopy (CLSM) (Figure 4D). The MTX-AMC-PEG nanoparticles were incubated with 4T1 cells for different times (2 h, 5 h, and 12 h). The blue fluorescence of nanoparticles was mainly in cytoplasm in the CLSM images, and the fluorescence intensity for 2 h incubation was much weaker than that for 5 h incubation. It implied that more nanoparticles were internalized in cells. The quantitative results of cellular uptake of the nanoparticles were tested by flow cytometry and the fluorescence intensity increased with prolongation of culture time, which indicated more MTX-ACM-PEG nanoparticles were accumulated in the cytosol.

## 4. Conclusions

In order to accelerate the MTX release from nanoparticles, a photo-responsive moiety was introduced in an amphiphilic MTX-AMC-PEG conjugate as the prodrug. The prodrugs self-assembled into nanoparticles and the drug release under laser irradiation was explored. The laser irradiation led to AMC photolysis and the cleavage of the ester bond in AMC moieties further destroyed the architecture of nanoparticles to trigger quick MTX release. This photo-sensitive release of MTX under laser irradiation exhibited excellent anticancer activity in vitro. This research potentially provides an effective strategy to fabricate a photo-responsive prodrug nanoparticle for chemotherapy.

## Supplementary Materials

The following are available online at https://www.mdpi.com/2079-4991/9/6/860/s1, Figure S1. ^1^H NMR of compounds after photolysis of MTX-ACM-PEG conjugate, (A) the compound of R_t_ at 2.57 min and (B) R_t_ at 7.85 min from HPLC; Figure S2. The size change of nanoparticles in different pH conditions (A), DLS curve of the nanoparticles in buffer solution after 10 h (B); Figure S3. The release profiles of nanoparticles in PBS (pH 7.4) with different concentration exposed to laser (365 nm, 5.0 W) for 1 h, (A) MTX-ACM-PEG conjugate in the solution; (B) the photocleavable release profiles of MTX from the nanoparticles. Means+SD (n = 3); Figure S4. The IC50 of the MTX and MTX-ACM-PEG nanoparticles against 4T1 cells, the incubation time was 48 h, the results were expressed as mean ± SD (n = 3); Figure S5. Compound **1** ^1^H NMR and ^13^C NMR spectra; Figure S6. Compound **2** ^1^H NMR and ^13^C NMR spectra; Figure S7. Compound **3** ^1^H NMR and ^13^C NMR spectra; Figure S8. Compound **4** ^1^H NMR spectra; Figure S9. IR spectra; Figure S10. Mass spectra.
