# Supplementary material for: A Photocleavable Amphiphilic Prodrug Self-Assembled Nanoparticles with Effective Anticancer Activity In Vitro
Source: Nanomaterials (Basel). 2019 Jun 5;9(6):860. doi: 10.3390/nano9060860 (PMC6630543; doi:10.3390/nano9060860)
Supplement: Supplementary file 1 [file nanomaterials-09-00860-s001.pdf]

A Photocleavable Amphiphilic Prodrug Self-Assembled Nanoparticles with Effective Anticancer Activity In Vitro

Ji Chen [1,†] Guotao Li [1,†] Qihong Liu [1] Yan Liang [2] Miaochang Liu [1,*] Huayue Wu [1] and Wenxia Gao [1,*]

[1] College of Chemistry and Materials Engineering, Wenzhou University, Wenzhou 325027, China
[2] Department of Pharmaceutics, School of Pharmacy, Qingdao University, Qingdao 266021, China
† These authors contributed equally.

Experimental Section

1. Cytotoxicity test

The 4T1 breast cancer cells (5×10^3 cells/mL) were harvested and seeded in 96-well plates with 100 μL mediums for 24 h incubation before the tests. MTX-AMC-PEG conjugate in culture mediums were added to the medium-removed 96-well plates with different concentrations and incubated for 48 h. The culture medium was removed and the wells were washed with PBS (pH = 7.4). CCK-8 in DMEM (10%) was added to each well. After the cells were incubated for additional 4 h, the cell viability was determined by measuring the absorption at 450 nm using a microplate reader (Thermo Scientific MK3).

2. In vitro anticancer activity

The 4T1 breast cancer cells (5×10^3 cells/mL) were harvested and seeded in 96-well plates with 100 μL medium for 24 h incubation before the tests. 4T1 breast cancer cells were incubated with MTX-AMC-PEG nanoparticles (a certain concentration of MTX 0.4 μg/mL) for 4 h and exposed to laser irradiation with a wavelength of 365 nm laser (5W) for 0, 0.5, 1, 2, and 3 min, respectively. After laser irradiation, the cells were incubated with media for 48 h. The culture medium was removed and the wells were washed with PBS (pH = 7.4). CCK-8 dilution in DMEM (10%) was added to each well. After the cells were incubated for additional 4 h, the cell viability was determined by measuring the absorption at 450 nm using a microplate reader (Thermo Scientific MK3).

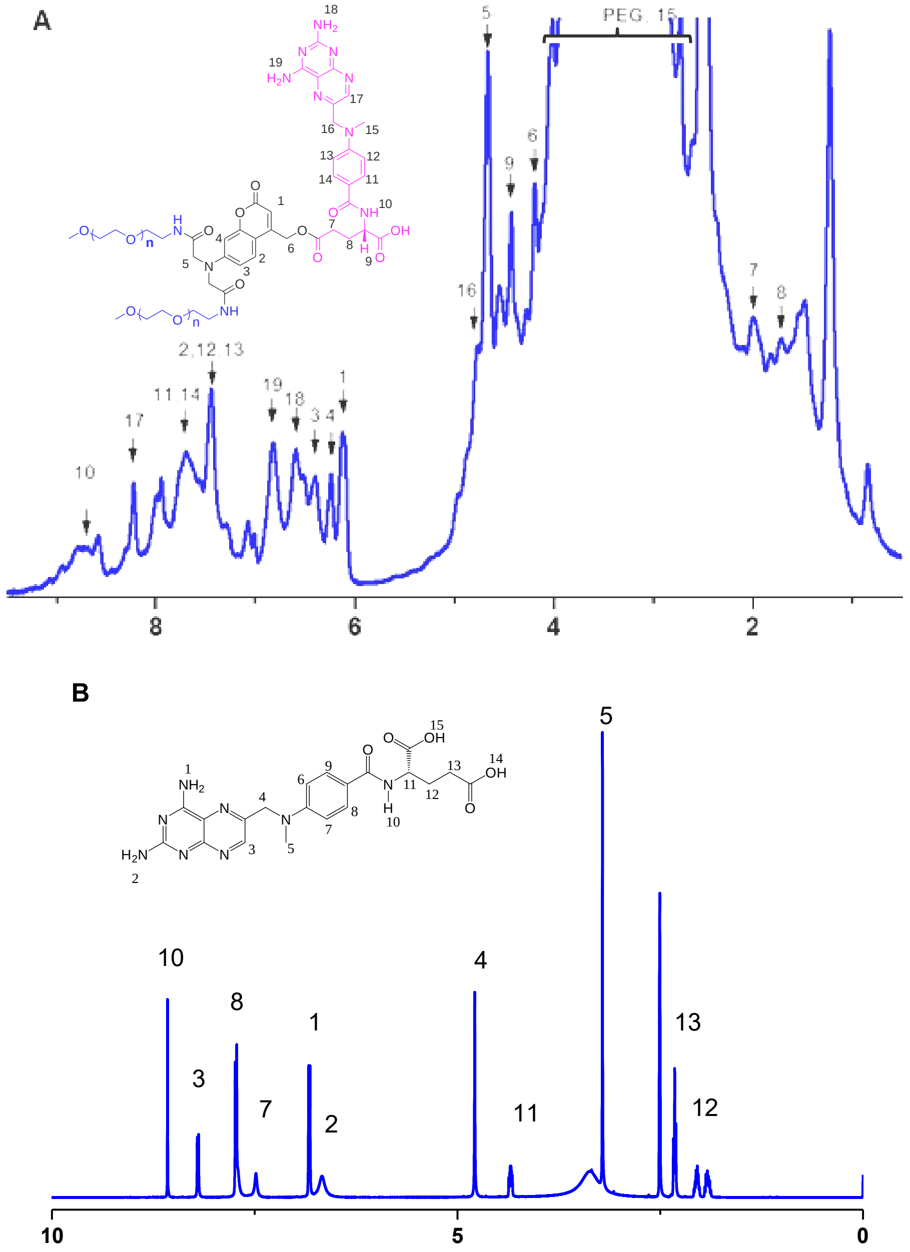

Figure S1. ^{1}H NMR of compounds after photolysis of MTX-ACM-PEG conjugate, (**A**) the compound of R_t at 2.57 min and (**B**) R_t at 7.85 min from HPLC.

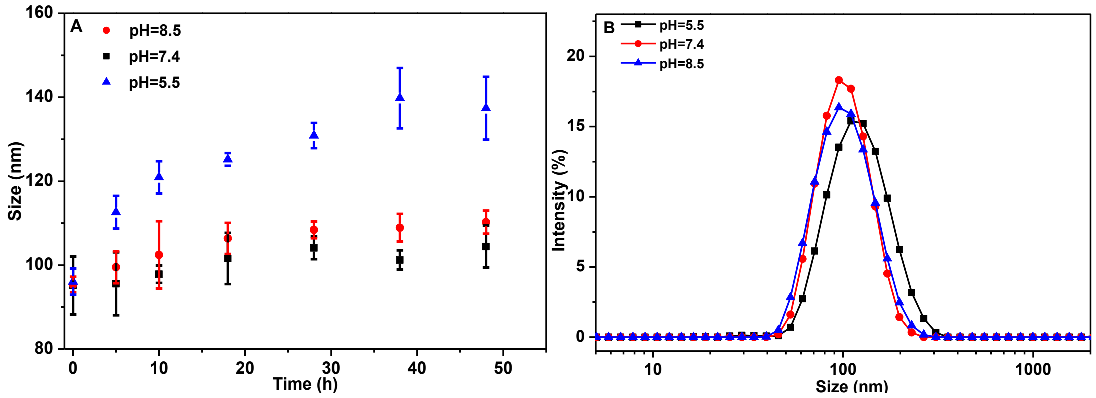

Figure S2. The size change of nanoparticles in different pH conditions (**A**), DLS curve of the nanoparticles in buffer solution after 10 h (**B**).

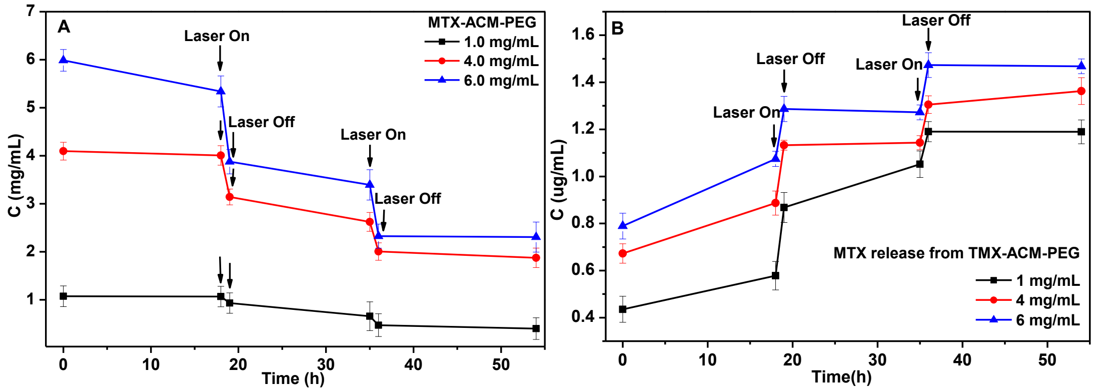

Figure S3. The release profiles of nanoparticles in PBS (pH 7.4) with different concentration exposed to laser (365 nm, 5.0 W) for 1 h, (**A**) MTX-ACM-PEG conjugate in the solution; (**B**) the photocleavable release profiles of MTX from the nanoparticles. Means+SD (n = 3).

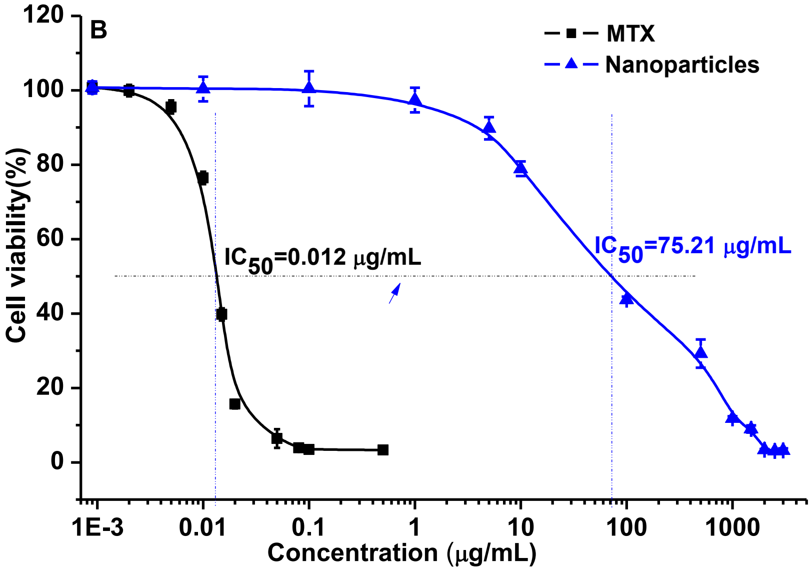

Figure S4. The IC50 of the MTX and MTX-ACM-PEG nanoparticles against 4T1 cells, the incubation time was 48 h, the results were expressed as mean ± SD (n = 3).

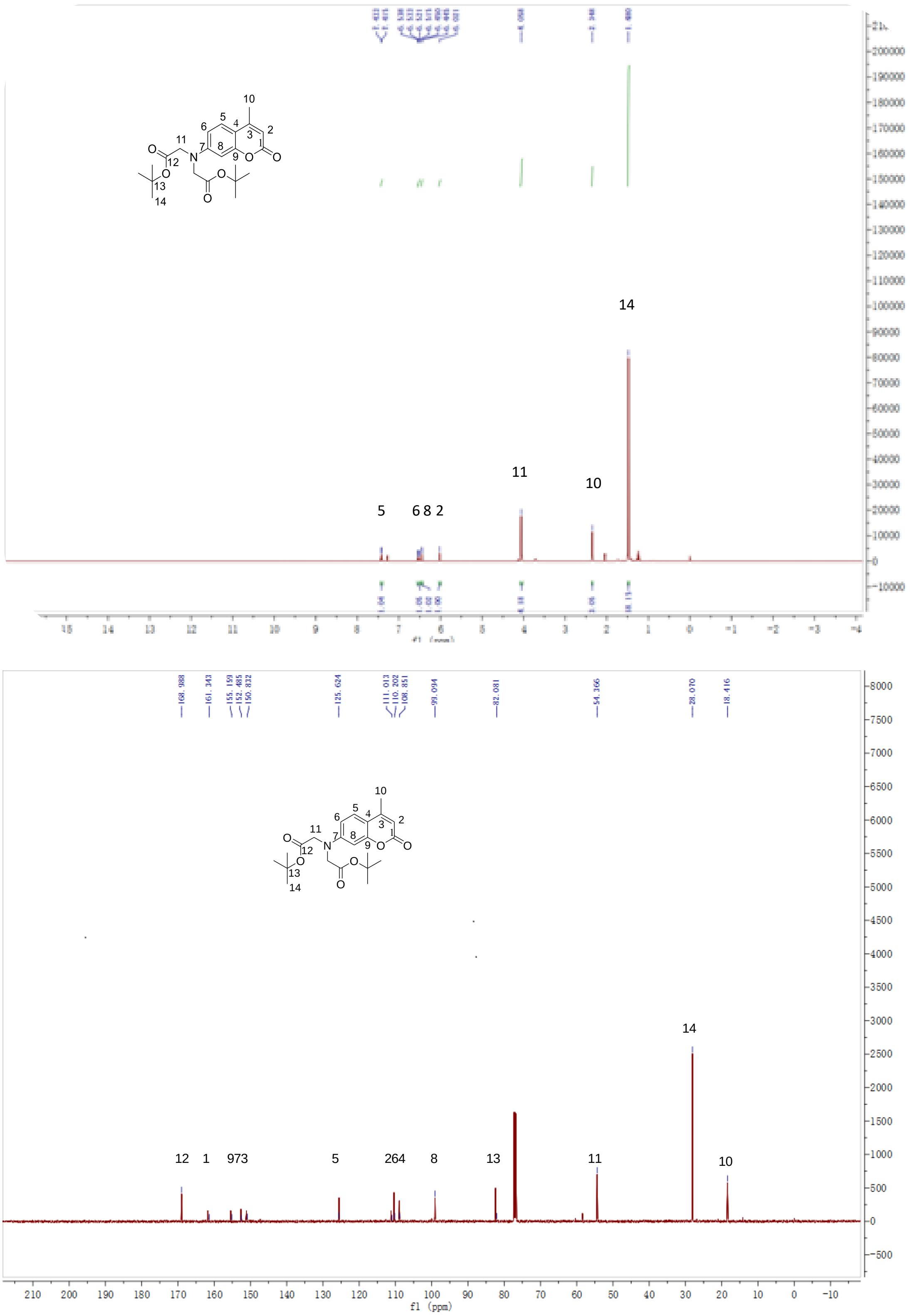

Figure S5. Compound **1** ^{1}H NMR and ^{13}C NMR spectra.

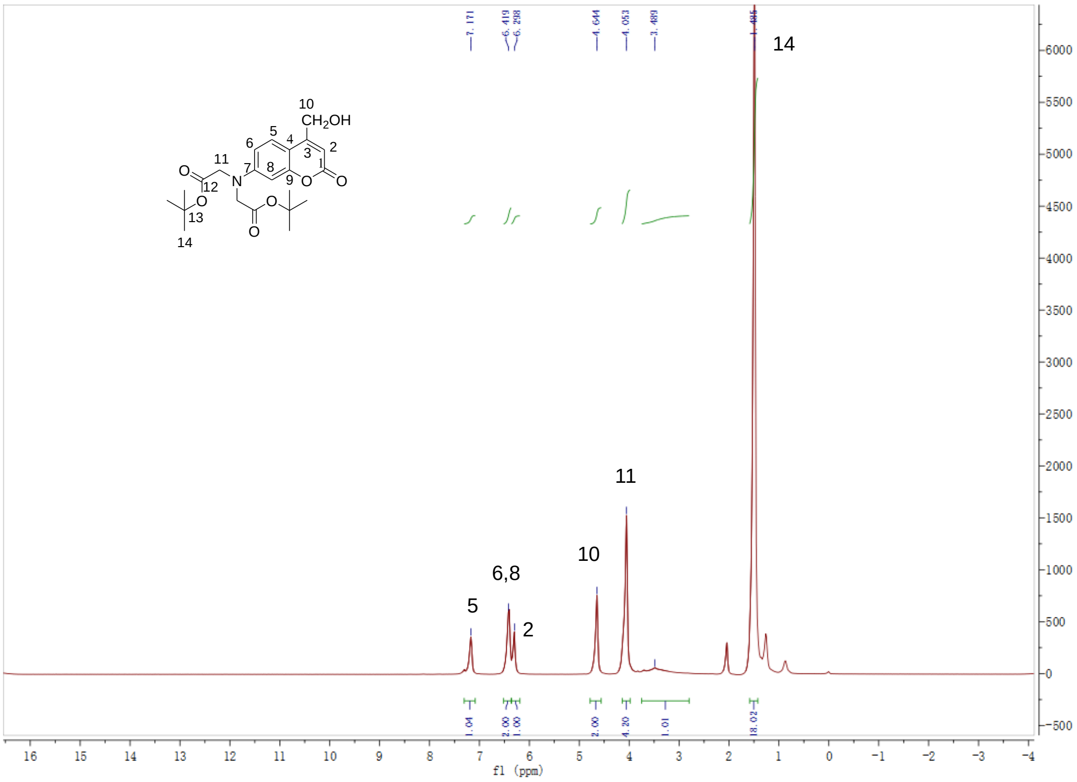

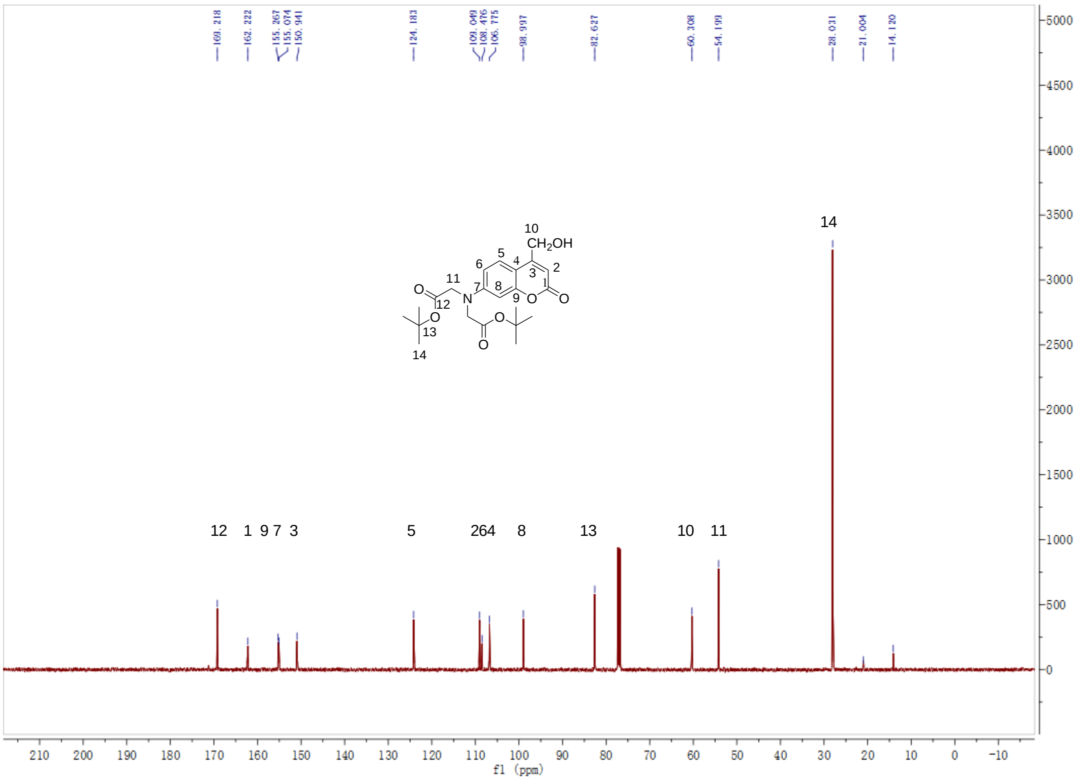

Figure S6. Compound **2** ^{1}H NMR and ^{13}C NMR spectra.

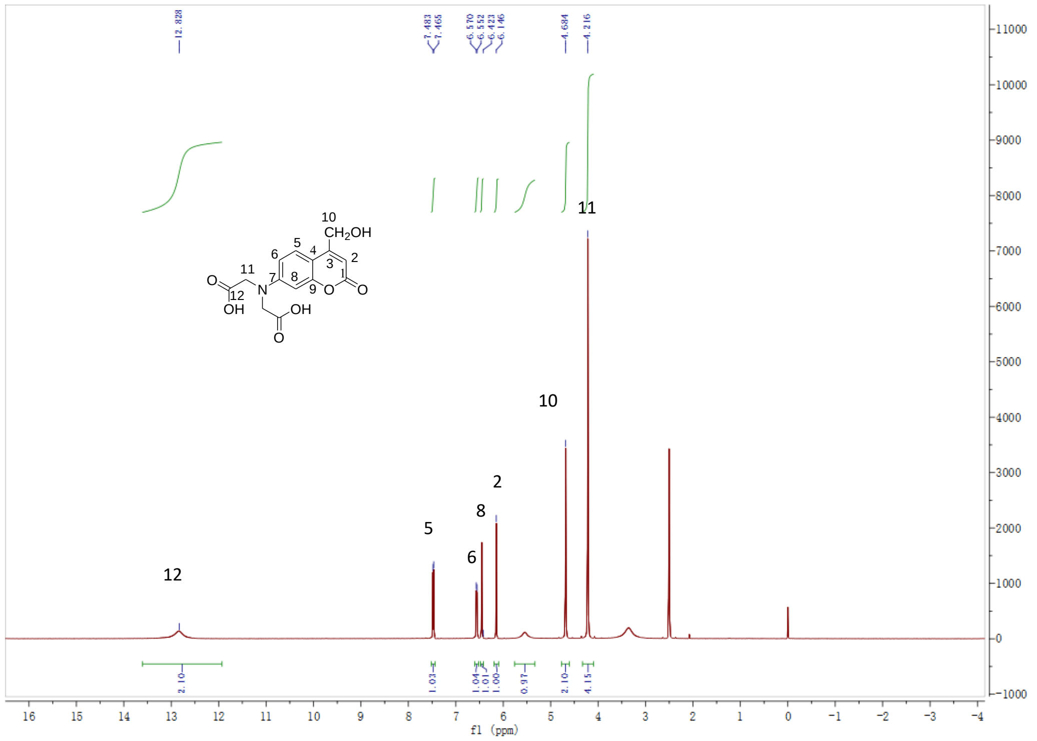

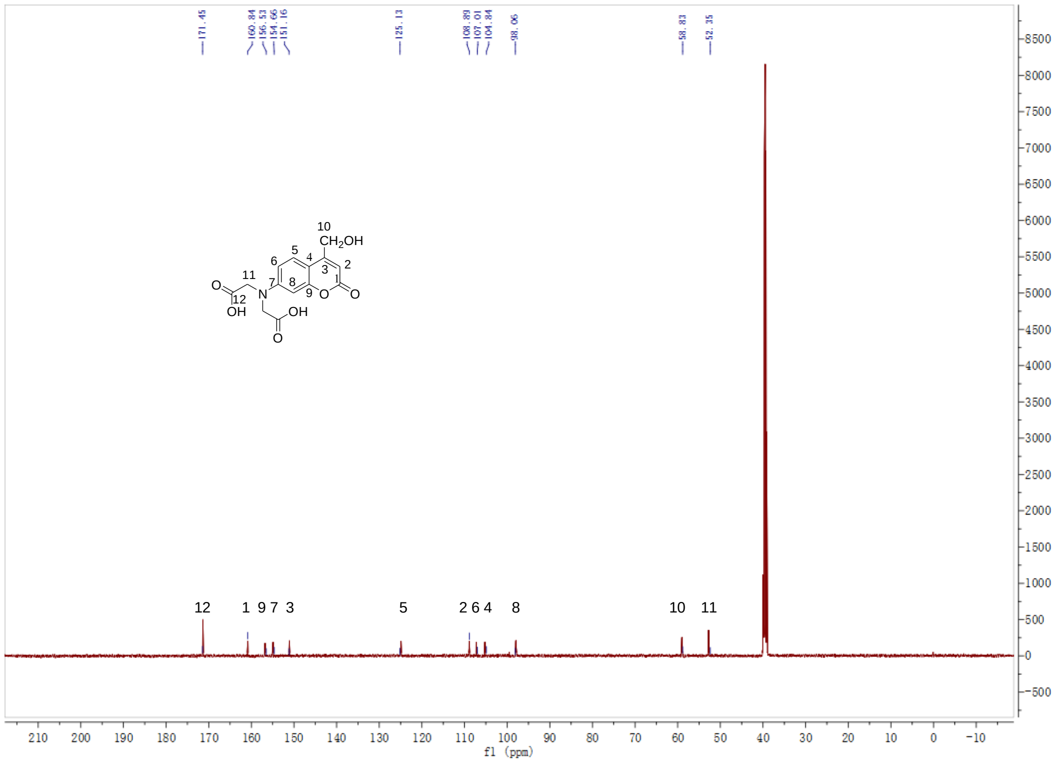

Figure S7. Compound **3** ^{1}H NMR and ^{13}C NMR spectra.

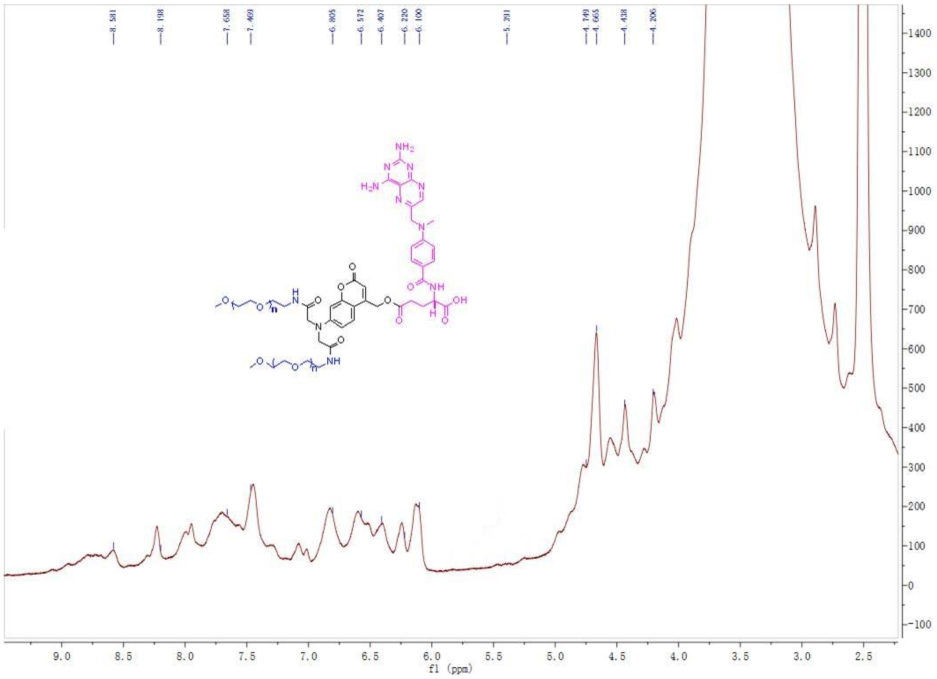

Figure S8. Compound **4** ^{1}H NMR spectra.

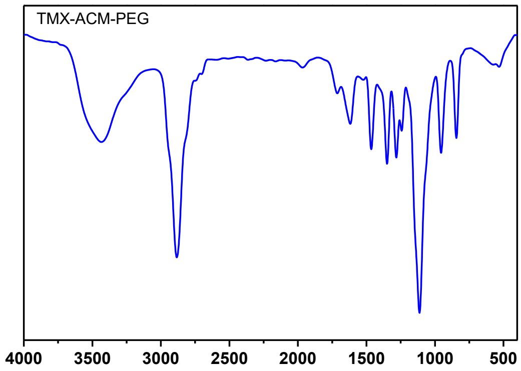

Figure S9. IR spectra.

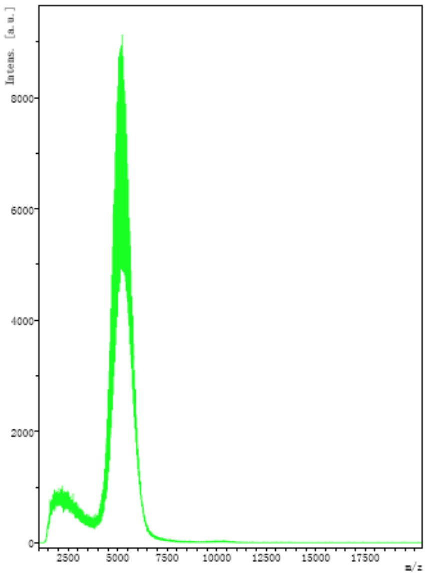

Figure S10. Mass spectra.
